# Supplementary material for: Predicting species invasiveness with genomic data: Is genomic offset related to establishment probability?
Source: Evol Appl. 2024 Jun 14;17(6):e13709. doi: 10.1111/eva.13709 (PMC11178484; doi:10.1111/eva.13709)
Supplement: Supplementary file 1 — Appendix S1. [file EVA-17-e13709-s001.docx]

# 991 A Supplementary Figures

1.0

0.5

Environmental/Phenotypic values

0.0

−0.5


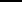
 Environment


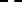
 Mean phenotype

Population
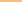
 Pop1
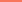
 Pop7
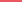
 Pop13
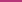
 Pop19
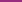
 Pop25

−1.0

0 1000 2000 3000

Generations

Figure S1: **Evidence of local adaptation in simulated native areas.** Evolution of environ- mental optimum 1 and mean realized phenotype along generations, for 5 populations of the native area simulations. A single random replicate is considered, for the scenario with low migration and environment type L. Populations indices are given in Figure 1.


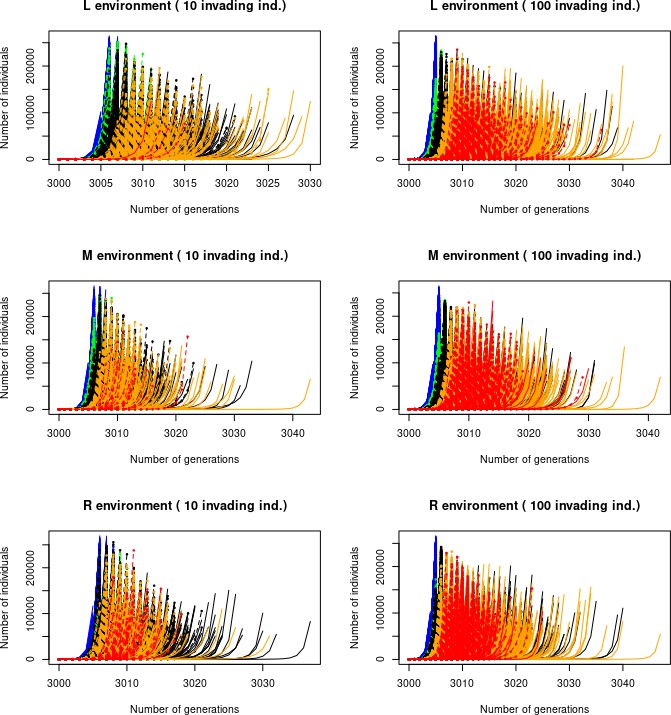

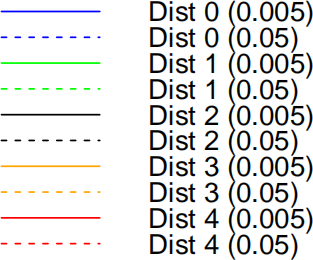


Figure S2: **Population size in the invaded area as a function of time (in generations)**, for the -1/-1 source population, across different environment types (L on the top, M in the middle and R in the bottom) and different numbers of invading individuals (10 on the right and 100 on the left). Populations always reach 50 000 individuals way before 100 simulated generations, and never face extinction after reaching this value. Colors correspond to the absolute environmental distance between the -1/-1 source population and the invaded environments (example : “Dist 2” includes the invaded environments at a distance of 2, either 0/0, 1/0 or 0/1). Solid lines represent the evolution of the number of individuals for low migration rate, and dashed lines for high migration rate.

Invaded environment


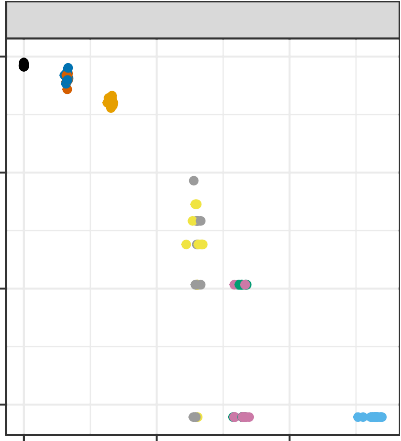


10 invading individuals

0

−2

−4

−6


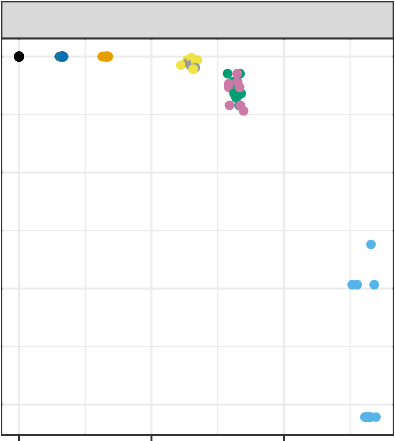


100 invading individuals


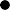
 −1/−1
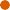
 −1/0


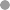
 −1/1
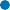
 0/−1

log(pe)


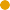
 0/0


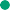
 0/1


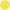
 1/−1


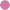
 1/0


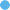
 1/1

0.000 0.005 0.010 0.000 0.005 0.010

gGOmc

Figure S3: **Evaluating the correlation between GO (here gGO_mc_) and log(*p_e_*)**. On each panel, log(*p_e_*) is plotted as a function of gGO_mc_ for 90 observations (10 repetitions of the native environment scenario *×* 9 possible invaded environments), which are combined to compute one R^2^ value. The left panel presents results for 10 invading individuals, and the right one for 100 invading individuals. In this example, the source population for the invasion is of -1/-1 type, migration rate is low, the native environment type is L, and only causal variables were used to compute gGO_mc_.

0.22


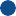

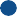

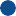

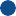

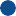

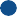

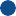

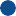

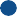

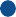

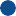

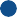

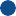

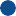

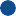

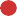

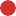

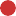

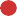

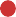

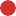

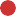

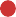

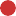

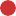

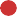

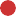

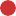


Heterozygosities estimates

0.21

Population status


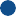
 Native


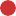
 Non−native

0.20

1 2 3 4 5 6 7 8 9 10 11 12 13 14 15 16 17 18 19 20 21 22 23 24 25 26 27 28

Population index

Figure S4: **Heterozygosities estimates computed with *poolfstat* for the 28 *B. tryoni***

### populations.

R^2^ between GO and log(EP) for low migration and 10 invading individuals

0.75

Linear

Mountain

Random

Causal var.

0.50

0.25

Mean R^2^ between GO and log(EP)

0.75

0.50

0.25

GO Method


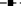
 Baypass (IS)
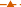
 Baypass (MC)
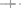
 Eucl. Distance
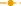
 GF

All var.


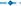
 LFMM

0.75

0.50

PCs

0.25

−1/−1 1/1 0/0 −1/−1 1/1 0/0 −1/−1 1/1 0/0

Invasion source

Figure S5: **Mean R**^2^ **values between GO computed on top 10% XtX* QTNs and log(*p_e_*) (for the low migration rate and 10 invading individuals) across the different native environment types (L on the left; M in the middle; and R on the left).** The results are presented as a function of the covariables included in the computation of GO (two causal variables on top; eight covariables including two causal and six confounding covariables on center; and five PCs at bottom). Each panel represents the mean R^2^ value over 90 observations for each of the 3 possible source population for invasion; specified on the x-axis; and over ten replicated simulation for the different GO estimators (GO_gf_, gGO_lfmm_, gGO_is_) alongside with Euclidean environmental distance.

R^2^ between GO and log(EP) for high migration and 10 invading individuals

0.75

Linear

Mountain

Random

Causal var.

0.50

0.25

Mean R^2^ between GO and log(EP)

0.00

0.75

0.50

0.25

0.00

GO Method


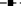
 Baypass (IS)
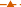
 Baypass (MC)
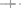
 Eucl. Distance
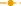
 GF

All var.


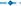
 LFMM

0.75

0.50

PCs

0.25

0.00

−1/−1 1/1 0/0 −1/−1 1/1 0/0 −1/−1 1/1 0/0

Invasion source

Figure S6: **Mean R**^2^ **values between GO computed on top 10% XtX* QTNs and log(*p_e_*), for high migration and 10 invading individuals.** Results are presented for different environment types (columns) and covariables included in the computation of GO (lines). Each panel represents the mean R^2^ value over 90 observations for each of the 3 possible source population for invasion; specified on the x-axis; and over ten replicated simulation for the different GO estimators (GO_gf_, gGO_lfmm_, gGO_is_ and gGO_mc_, see the main text for details) alongside with Euclidean environmental distance.

R^2^ between GO and log(EP) for high migration and 10 invading individuals

0.75

Linear

Mountain

Random

Causal var.

0.50

0.25

Mean R^2^ between GO and log(EP)

0.00

0.75

0.50

0.25

0.00

GO Method


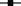
 Baypass (IS)
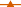
 Baypass (MC)
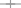
 Eucl. Distance
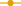
 GF

All var.


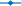
 LFMM

0.75

0.50

PCs

0.25

0.00

−1/−1 1/1 0/0 −1/−1 1/1 0/0 −1/−1 1/1 0/0

Invasion source

Figure S7: **Mean R**^2^ **values between GO computed with all QTNs and log(*p_e_*), for high migration and 10 invading individuals.** See Figure S6 for more details.

R^2^ between GO and log(fitness) for low migration and 10 invading individuals

1.00

Linear

Mountain

Random

0.75

Causal var.

0.50

Mean R^2^ between GO and log(fitness)

0.25

0.00

1.00

0.75

0.50

0.25

0.00

1.00

GO Method


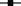
 Baypass (IS)
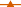
 Baypass (MC)
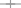
 Eucl. Distance
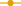
 GF

All var.


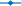
 LFMM

0.75

0.50

PCs

0.25

0.00

−1/−1 1/1 0/0 −1/−1 1/1 0/0 −1/−1 1/1 0/0

Invasion source

Figure S8: **Mean R**^2^ **values between GO computed with all QTNs and log(*fitness*), for low migration and 10 invading individuals.** See Figure S6 for more details.

R^2^ between GO and log(GR) for low migration and 10 invading individuals

1.00

Linear

Mountain

Random

0.75

Causal var.

0.50

0.25

Mean R^2^ between GO and log(GR)

1.00

0.75

0.50

0.25

1.00

GO Method


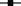
 Baypass (IS)
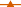
 Baypass (MC)
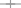
 Eucl. Distance
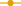
 GF

All var.


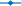
 LFMM

0.75

0.50

PCs

0.25

−1/−1 1/1 0/0 −1/−1 1/1 0/0 −1/−1 1/1 0/0

Invasion source

Figure S9: **Mean R**^2^ **values between GO computed on all QTNs and log(*growthrate*), for high migration and 10 invading individuals.** See Figure S6 for more details.

R^2^ between GO and log(fitness) for low migration and 100 invading individuals

1.00

Linear

Mountain

Random

0.75

Causal var.

0.50

Mean R^2^ between GO and log(fitness)

0.25

1.00

0.75

0.50

0.25

1.00

GO Method


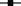
 Baypass (IS)
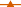
 Baypass (MC)
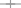
 Eucl. Distance
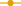
 GF

All var.


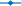
 LFMM

0.75

0.50

PCs

0.25

−1/−1 1/1 0/0 −1/−1 1/1 0/0 −1/−1 1/1 0/0

Invasion source

Figure S10: **Mean R**^2^ **values between GO computed on all QTNs and log(*fitness*), for low migration and 100 invading individuals.** See Figure S6 for more details.

0.006


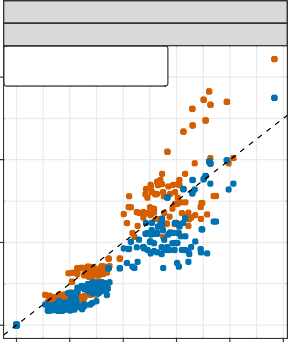


Low migration

Linear

MAPE Baypass = 22 % MAPE LFMM = 34 %

0.0015


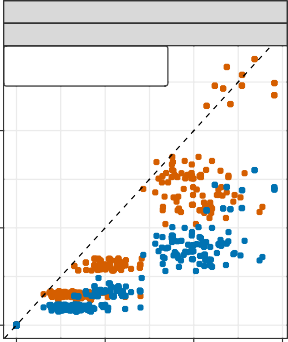


Low migration

Mountain

MAPE Baypass = 37 % MAPE LFMM = 64 %


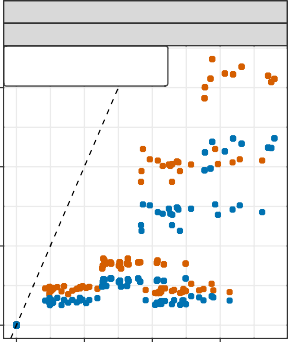


Low migration

Random

MAPE Baypass = 69 % MAPE LFMM = 78 %

0.004

0.002

0.0010

0.002

0.001

0.0005

0.000

GGO computed on all QTNs

0.000

0.0000

0.000 0.001 0.002 0.003 0.004 0.005 0.000 0.001 0.002 0.003 0.000 0.001 0.002 0.003


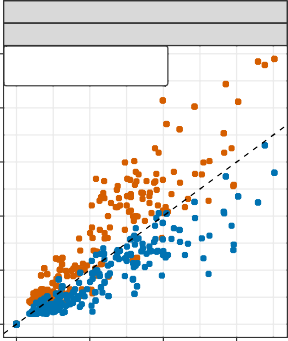
High migration Mountain


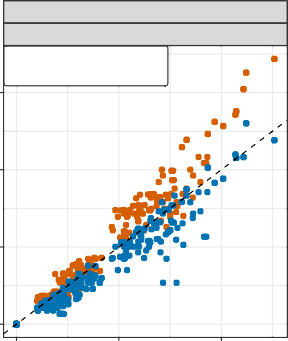


High migration

Linear

MAPE Baypass = 19 % MAPE LFMM = 17 %


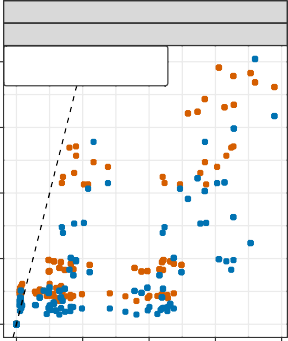


High migration

Random

MAPE Baypass = 77 % MAPE LFMM = 81 %

GGO Method


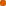
 Baypass (MC)
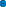
 LFMM

0.0015

5e−04 MAPE Baypass = 47 %

MAPE LFMM = 26 %

0.00020

4e−04

0.00015

0.0010

3e−04

2e−04

0.00010

0.0005

1e−04

0.00005

0.0000

0e+00

0.00000

0e+00 5e−04 1e−03 0e+00 1e−04 2e−04 3e−04 0e+00 2e−04 4e−04 6e−04 8e−04

F2 computed on all QTNs

Figure S11: **Comparison of gGO_mc_ and gGO_lfmm_ with *f***_2_ **statistics**, for different native environment types (columns) and migration rates (lines). All causal QTNs were used for gGO and *f*_2_ computation, without applying a MAF filter and excluding neutral markers. MAPE is indicated for each method and each type of scenario. The R environment dataset has fewer data points due to the absence of certain environmental value combinations in the native grid, resulting from the random selection of these values.

0.006


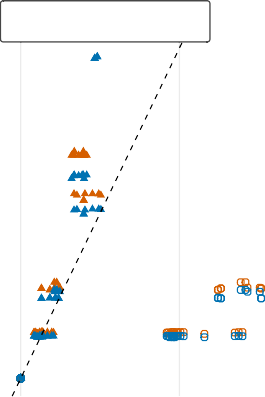


MAPE Baypass = 49 / 77 % MAPE LFMM = 38 / 79 %

Mountain (M)

Low migration

Low migration Random (R)

MAPE Baypass = 48 / 90 %

MAPE LFMM = 62 / 120 %

0.004

0.004

0.002

0.002

gGO computed on all markers (QTNs + Neutral)

0.000

9e−04

6e−04

3e−04

0e+00

0.000

0.000 0.005 0.010 0.00 0.01 0.02 0.03

| High migration | | | | | | |
| --- | --- | --- | --- | --- | --- | --- |
| Mountain (M) | | | | | | |
| MAPE Bayp | ass = 208 / 45 | % |  |  |  |  |
| MAPE LFM | M = 217 / 44 | % |  |  |  |  |
|  |  |  |  |  |  |  |
|  |  |  |  |  |  |  |
|  |  |  |  |  |  |  |
|  |  |  |  |  |  |  |
|  |  |  |  |  |  |  |
|  |  |  |  |  |  |  |
|  |  |  |  |  |  |  |

| High migration | | | | |
| --- | --- | --- | --- | --- |
| Random (R) | | | | |
| MAPE Baypass  MAPE LFMM | = 135 / 944 %  = 150 / 1007 % |  |  |  |
|  |  |  |  |  |
|  |  |  |  |  |
|  |  |  |  |  |
|  |  |  |  |  |
|  |  |  |  |  |
|  |  |  |  |  |
|  |  |  |  |  |
|  |  |  |  |  |

0.0012


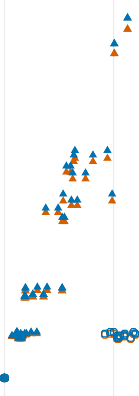

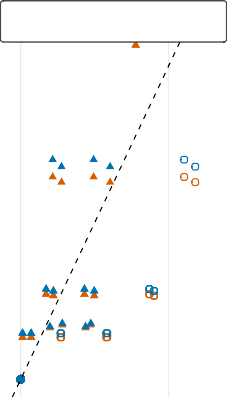


0.0009

0.0006

0.0003

0.0000

0e+00 3e−04 6e−04 9e−04 0.000 0.001 0.002

F2

F2 computation


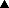
 Causal F2


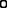
 QTN+Neut F2

gGO Method


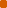
 Baypass (MC)
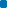
 LFMM

Figure S12: **Comparison of gGO computed using BayPass (MC) or LFMM with *f***_2_ **statis- tics derived from either QTNs alone or QTNs and Neutral markers (MAF-filtered), for M and R environments**. gGO computations involved QTNs and Neutral markers (MAF filtered). MAPE is indicated for each method and each type of scenario. The R environment dataset has fewer data points due to the absence of certain environmental value combinations in the native grid, re- sulting from the random selection of these values.


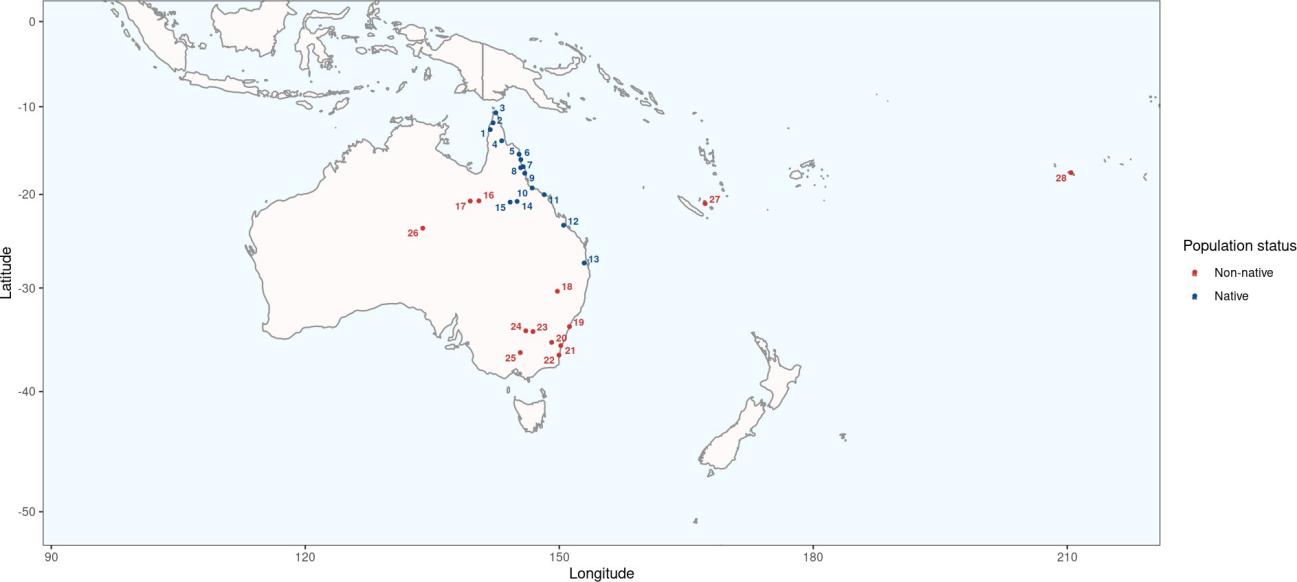


Figure S13: **Geographical Distribution of 28 *B. tryoni* populations studied by Popa-B´aez *et al.* (2020)**. Populations 1 to 15 (in blue) are native populations, while populations 16 to 28 (in red) established outside the species’ native range at different time points during the second half of the 20th century.

200


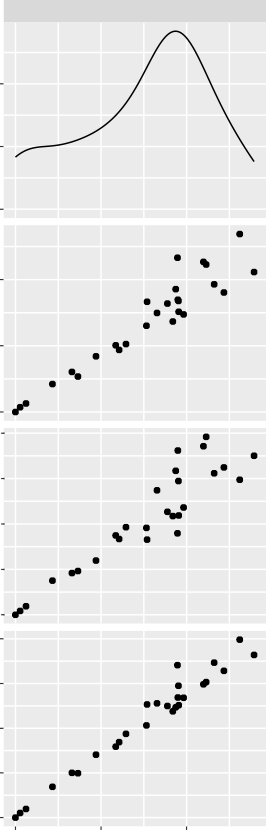

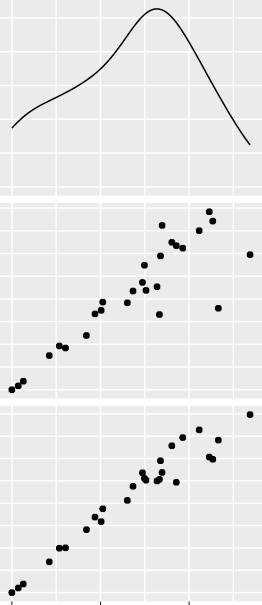

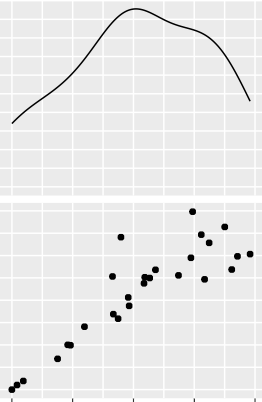


LFMM

Baypass.MC

GF

Eucl.Dist

Corr:

0.910***

Corr:

0.905***

Corr:

0.955***

Corr:

0.858***

Corr:

0.951***

Corr:

0.849***

LFMM

100

0

0.004

0.002

Baypass.MC

0.000

0.00100

0.00075

0.00050

GF

0.00025

0.00000

20

15

10

Eucl.Dist

5

0

0.000 0.002 0.004 0.000 0.002 0.004 0.00000 0.00025 0.00050 0.00075 0.00100 0 5 10 15 20

Figure S14: **Correlation between gGO_mc_, gGO_lfmm_, GO_gf_ and Euclidean distance at the location of the 27 populations of *B. tryoni* , computed with PCs.** The population 28 is not taken into account because its GO values were not computed due to Tahiti being too far from the Australian mainland. The lower panels display the correlations between pairs of measures, while the upper panels present the Spearman correlation coefficients for each pair of GO measures. The significance of the p-value for the Spearman correlation test is denoted by stars (*** when p-value

*<* 0.001), and the diagonal exhibits the distribution of each GO measure.

**gGOlfmm gGOlfmm (univariate)**


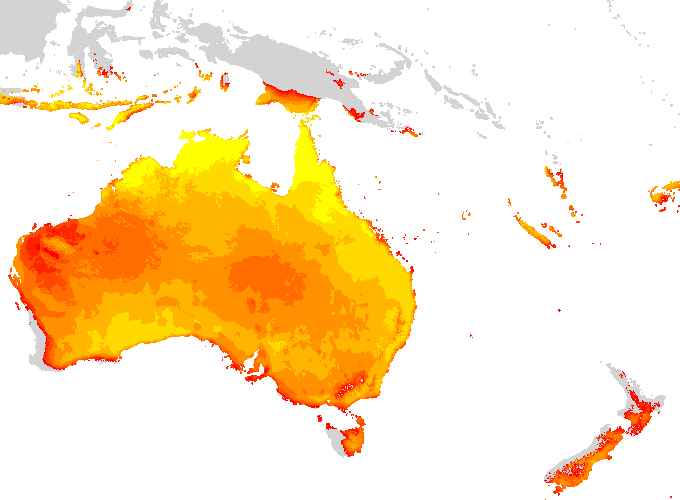


0

0

- 1. e+00

−10

1.4e−02

1.2e−02

−20

1.0e−02

8.5e−03

6.8e−03

- 1. e−03

−30

3.4e−03

- 1. e−03

0.0e+00

−40

4.3e−01

- 1. e−02

−10

- 1. e−02

−20

1.4e−02

- 1. e−02

9.2e−03

6.9e−03

−30

4.6e−03

2.3e−03

0.0e+00

−40

100 120 140 160 180 100 120 140 160 180

**gGOmc gGOis**

0

0

- 1. e+00

−10

1.4e−02

1.2e−02

−20

1.0e−02

8.6e−03

6.9e−03

- 1. e−03

−30

3.4e−03

- 1. e−03

0.0e+00

−40

7.9e−03

5.2e−04

−10

4.6e−04

−20

3.9e−04

3.3e−04

2.6e−04

2.0e−04

−30

- 1. e−04

6.5e−05

0.0e+00

−40

100 120 140 160 180 100 120 140 160 180

**GOgf**

0

**Euclidean distance**

0

5.4e−02

−10

1.6e−03

- 1. e−03

−20

1.2e−03

9.7e−04

7.8e−04

5.8e−04

−30

3.9e−04

1.9e−04

0.0e+00

−40

5.7e+02

3.9e+01

−10

3.4e+01

−20

2.9e+01

2.4e+01

1.9e+01

1.5e+01

−30

9.7e+00

4.9e+00

0.0e+00

−40

100 120 140 160 180 100 120 140 160 180

Figure S15: **GO estimated between population 1 (“source” population), and a large area in Oceania, with gGO_lfmm_, gGO_lfmm_ modified in ordered to treat variables indepen- dently, gGO_mc_, gGO_is_ and GO_gf_, using six variables for GO computation (bio** **3 , bio** **5, bio8, bio** **9 and bio** **12).** Squared Euclidean distance to the source population is also displayed. Grey pixels represent outliers values.

200

LFMM

LFMM..univariate.

Baypass.MC

Baypass.IS

GF

Euclidean.distance

Corr:

0.645***

Corr:

0.991***

Corr:

0.638***

Corr:

0.848***

Corr:

0.844***

Corr:

0.659***

Corr:

0.976***

Corr:

0.861***

Corr:

0.732***

Corr:

0.653***

Corr:

0.866***

Corr:

0.852***

Corr:

0.868***

Corr:

0.687***

Corr:

0.801***

150

100

LFMM

50

0

0.015

LFMM..univariate.

0.010

0.005

0.000

0.008

0.006

Baypass.MC

0.004

0.002

0.000

4e−04

3e−04

Baypass.IS

2e−04

1e−04

0e+00

0.00100

0.00075

0.00050

GF

0.00025

0.00000

20

15

Euclidean.distance

10

5

0

0.000 0.002 0.004 0.006 0.000 0.005 0.010 0.015 0.000 0.002 0.004 0.006 0.008 0e+00 1e−04 2e−04 3e−04 4e−004.00000 0.00025 0.00050 0.00075 0.00100 5 10 15 20

Figure S16: **Correlation between gGO_lfmm_, gGO_lfmm_ (univariate), gGO_mc_, gGO_is_, GO_gf_ and Euclidean distance at the location of the 27 populations of *B. tryoni* , computed on six selected variables (bio3 , bio5, bio8, bio** **9 and bio** **12).** The population 28 is not taken into account because its GO values were not computed due to Tahiti being too far from the Australian mainland. The lower panels display the correlations between pairs of measures, while the upper panels present the Spearman correlation coefficients for each pair of GO measures. The significance of the p-value for the Spearman correlation test is denoted by stars (*** when p-value

*<* 0.001), and the diagonal exhibits the distribution of each GO measure.

0/0 source population

0

−2

−4

−6

0.000 0.005 0.010 0.000 0.005 0.010

log(p_e_)

gGO_mc_

Invaded environment

−1/−1 −1/0

−1/−1 source population

−1/1 0/−1

0/0

0/1

1/−1

1/0

1/1

Figure S17: **Comparison between 0/0 and -1/-1 source populations concerning the corre- lation between GO (here gGO_mc_) and log(*p_e_*)**. On each panel, log(*p_e_*) is plotted as a function of gGO_mc_ for 90 observations (10 repetitions of the native environment scenario *×* 9 possible invaded environments), which are combined to compute one R^2^ value. The left panel presents results for 0/0 source population, and the right one for -1/-1 source population. In this example, migration rate is low, there are 10 invading individuals, the native environment type is L, and only causal variables were used to compute GO.

# 992 B Supplementary Table

| **Migration rate** | **Environment type** | **Mean nb. of QTNs** | **Mean nb.**  **of QTNs**  **(MAF 1%)** | **Mean nb. of**  **neutral mut.**  **(MAF 1%)** | **Mean fitness** | **Mean Fst** |
| --- | --- | --- | --- | --- | --- | --- |
|  | Linear | 1507 ± 35 | 44 ± 5 | 11 860 ± 112 | 0.952 ± 0.001 | 0.034 |
| 0.005 | Mountain | 1568 ± 29 | 64 ± 10 | 11 781 ± 116 | 0.933 ± 0.002 | 0.049 |
|  | Random | 1535 ± 24 | 62 ± 5 | 11 873 ± 95 | 0.945 ± 0.001 | 0.072 |
|  | Linear | 1756 ± 33 | 82 ± 12 | 11 548 ± 126 | 0.883 ± 0.002 | 0.0034 |
| 0.05 | Mountain | 1955 ± 43 | 109 ± 11 | 11 504 ± 61 | 0.807 ± 0.002 | 0.0040 |
|  | Random | 1852 ± 48 | 80 ± 10 | 11 509 ± 142 | 0.815 ± 0.002 | 0.0046 |

Table S1: **Information about native area simulations.** Means are computed over 10 replicates for each native area, and at the end of the 3,000 simulated generations. The standard deviation of each mean is displayed after ± sign.

# Supplementary Text

## Note S1: details on simulated scenarios

### Fitness

The fitness of an individual *i* living in the population *j* will increase as the cumulative effect sizes of its QTNs (representing its phenotype P_1_*_,ij_* and P_2_*_,ij_*) approach the values of the two environmental optima of the population *j* at the generation *t* (Θ_1_*_jt_* and Θ_2_*_jt_*). Individual fitness (*ω_ij_*) is computed using a multivariate normal distribution with a standard deviation of *σ_k_* as described in L´aruson *et al.* (2022) previous work :

*−*1 " *P*1*,ij −* Θ1*jt* 2 *P*2*,ij −* Θ2*jt* 2#

*ω_ij_* = *e* 2

+

(1)

*σ*

*k*

*σ*

*k*

where *σ_k_* reflects the intensity of stabilizing selection. Similar to the environmental optima Θ_1_*_jt_* and Θ_2_*_jt_*, *σ_k_* varied along the simulation of the native area. It was initially set at 4 during the first 1000 generations, implying weak stabilizing selection, enabling high variance in individual’s phenotypic values. It then gradually decreased over the next 1000 generations, ultimately reaching a value of

1.25 implying stronger stabilizing selection, which was kept constant during the final 1000 simulated generations.

### Life cycle and evolutionary rates

In the native area, evolution was simulated under a Wright-Fisher model, where generations did not overlap and population size was held constant. However, reproduction was not random : the fitness of individuals corresponded to their probability of being chosen as parents for the next generation, which favored the spread of locally advantageous mutations.

In the invaded area, the simulation framework diverges from the Wright-Fisher model to allow for population extinction, essential for calculating establishment probabilities. The simulation encom- passes three age classes, and includes senescence, with individuals beyond the first age class (adults) experiencing a halving of their fitness each generation. Individuals moved to the next age class when they survived, which occurred with a probability depending on their fitness. Details on life cycle are provided in Figure T1.

Native area :

Generation count increment :

Only offspring with age 0 survived to reach age 1, and can become parents.

Reproduction :

Offspring are generated from 1000 males and 1000 females selected as parents. Sampling is proportional to fitness, with individuals chosen with replacement. Each couple produces one offspring.

Fitness computation : Fitness of individuals are computed (see Eq. 1)

Survival :

Parents die (non overlapping generations)

Invaded area :

Generation 3000 : Start of the invasion

Generation count increment :

Surviving individuals have their age incremented

Reproduction :

A male is randomly sampled for each female (with the possibility of multiple samplings for a single male). Offspring are born, with the number of offspring for each couple drawn from a zero-inflated Poisson distribution.

Fitness computation : Fitness of individuals are computed (see Eq. 1).

Individuals aged ≥1 have their fitness halved.

Individuals aged = 3 have their fitness set to 0.

Survival : Survival is determined by random draws based on

fitness (=probability of survival); individuals aged 3 are certain not to survive.

### Figure T1: Simulation life cycle in the native (top panel) and invaded area (bottom panel).

These life cycle parameters were chosen to approximate the biology of invasive insects’, with charac- teristics such as high fecundity, short lifespan, fast development and overlapping generation (Sakai *et al.*, 2001; Zhao *et al.*, 2023). To simulate short lifespan and fast development, the majority of individuals experience only two age classes, one as juveniles and one as adults. To account for indi- viduals living beyond the average lifespan, their fitness is halved upon reaching the second age class. An important decrease in survival rate when population grows older has been reported in several invasive species, such as *Drosophila suzukii* (Tochen *et al.*, 2014), *B. tryoni* (Fanson and Taylor, 2012) or *Bactrocera dorsalis* (Jaleel *et al.*, 2018a).

Only adults can reproduce, with a 50% probability for a mating pair to produce offspring, consider- ing the possibility of non-successful mating. The number of offsprings for successful mating is drawn from a Poisson distribution, with an average of 20 offspring per mating pair. This number may seem low considering that for some species such as *D. suzukii*, or *Bactrocera* species, the fecundity (=Lifetime production of offspring (eggs) by an average female that lives to the last day of life in the cohort) can sometimes reach more than 500 eggs produced (Hamby *et al.*, 2016; Jaleel *et al.*, 2018b). However, preliminary tests were conducted, exploring mean offspring numbers of 5, 20, and 100 per mating pair, and they indicated that simulation results remained relatively stable.

It’s important to note that while these life cycle parameters were designed to align with the biology of insect invasive species, they can vary significantly depending on factors such as humidity, tem- perature, rearing conditions, population density, and more (Hamby *et al.*, 2016). Consequently, the

simulations were not intended to precisely replicate the life cycle of any specific species. Instead, the primary objective was to construct a simulation model that captures the general characteristics of an insect-like species.

## Note S2: Genetic Offset measures

### GO computation

**GF correction with LFMM residuals.** LFMM are regression models which can be written as

follows :

*Y* = *XB^T^* + *W* + *E* (2)

When studying n populations for p genetic markers, Y represents the n *×* p response matrix of centered allele frequencies. As explained in Caye *et al.* (2019), “the fixed effect sizes are recorded in the B matrix, which has dimension p *×* d”, where d represents the total number of primary and nuisance variables. “The E matrix represents residual errors, and it has the same dimensions as the response matrix. The matrix W is a latent matrix of rank K, defined by K latent factors.” By performing a Singular Value Decomposition (SVD) of the W matrix, one can obtain U and V matrix, corresponding respectively to the confounding factors matrix, and to the loadings of these factors :

*W* = *UV ^T^* (3)

W matrix can then be subtracted from Y matrix as shown in equation (4), in order to obtain the residuals of the model, representing the corrected centered allele frequencies.

*Y − W* = *XB^T^* + *E* (4)

**Optimized GF.** For GF analyses, a modified version of *Gradient Forest* v 0.1.32 package was used. Indeed, this package was developed to analyze species abundance data and is not optimized for handling very large datasets such as those used in population genomics. Therefore, we have optimized the package to be able to create models using large datasets, significantly reducing computation times and memory footprint, while maintaining very similar predicted GO values (Figure T2). Note that this adapted package is (so far) compatible exclusively with continuous predictors, not with discrete ones. The resulting R function is available for download at https://forgemia.inra.fr/simon. boitard/popgenomicprediction

**Computation Time Memory Peak**

700

40

600

35 500

Mem. Peak (in MiB)

400

Time (in s)

30

300

200

25

100

20

GF_opt GF0 GF_opt GF0

**Predicted GO values**

GF0

0.015

0.020

0.025

0.000 0.005 0.010 0.015 0.020 0.025

0.000

0.005

0.010

GF_opt

Figure T2: Comparison of performance metrics (Computation Time and Memory Use) and predicted GO values between the original Gradient Forest package (GF0) and the modified version (GF opt). Tests were run with 10 different seeds.

## Note S3 : Variable importances with PCs

Let ***E*** = ***UDV ^′^*** the singular value decomposition of the matrix of the original *D* (centered) covari- able values for the *J* populations. By construction, the eigenvector matrix ***U*** is the scaled matrix of the *P* principal components (PCs) (i.e., ***U^′^U*** = ***I***); the diagonal matrix ***D*** contains the square root of the eigenvalues in its diagonal; and the orthogonal matrix ***V*** contains the covariable loadings of each PC (with ***V ^′^V*** = ***I***).

From this original decomposition, it is then easy to rescale the matrix ***B_I×P_*** of the regression coefficients estimated for each of the *I* SNPs and associated to each of the *P* PCs to obtain a *I × D* matrix ***C*** of regression coefficients associated to each of the original covariables. Indeed, under linear model assumptions underlying the BayPass (or LFMM) GEA models, the expectation of the vector ***α_i_*** of allele frequencies in the *J* populations may be written as:

E (***α_i_***) = *µ_i_***1***_J_* + ***b^′^ U***

***i***

where ***b_i_*** is a *P* length vector corresponding to the *i*th row of the regression coefficient matrix ***B***. Hence:

E (***α_i_***) = *µ_i_***1***_J_* + ***Ub_i_***

= *µ_i_***1***_J_* + ***EV D^−^*^1^** ***b_i_***

= *µ_i_***1***_J_* + ***E*** ***V D^−^*^1^*b_i_***

The *D* length vector ***c_i_*** = ***V D^−^*^1^*b_i_*** mays thus be interpreted as the regression coefficients associated to each original covariable. Hence the *I × D* matrix ***C*** can simply be obtained as:

***C*** = ***V D^−^*^1^*B^′^*** *′*

This matrix can then be decomposed to derive the importance of each covariable.

**REFERENCES**

Fanson, B. G., & Taylor, P. W. (2012). Additive and interactive effects of nutrient classes on longevity, reproduction, and diet consumption in the Queensland fruit fly (*Bactrocera tryoni*). *Journal of Insect Physiology*, *58*(3), 327–334.

Hamby, K. A., Bellamy, E. D., Chiu, J. C., Lee, J. C., Walton, V. M., Wiman, N. G., York, R. M., & Biondi, A. (2016). Biotic and abiotic factors impacting development, behavior, phenology, and reproductive biology of Drosophila suzukii. *Journal of Pest Science*, *89*(3), 605–619.

Jaleel, W., Tao, X., Wang, D., Lu, L., & He, Y. (2018). Using two‐sex life Table traits to assess the fruit preference and fitness of *Bactrocera dorsalis* (Diptera: Tephritidae). *Journal of Economic Entomology*, *111*(6), 2936–2945.

Jaleel, W., Yin, J., Wang, D., He, Y., Lu, L., & Shi, H. (2018). Using two‐sex life tables to determine fitness parameters of four Bactrocera species (Diptera: Tephritidae) reared on a semi‐artificial diet. *Bulletin of Entomological Research*, *108*(6), 707–714.

Tochen, S., Dalton, D. T., Wiman, N., Hamm, C., Shearer, P. W., & Walton, V. M. (2014). Temperature‐related development and population parameters for *Drosophila suzukii* (Diptera: Drosophilidae) on cherry and blueberry. *Environmental Entomology*, *43*(2), 501–510.
